# The Production of a Stable Infliximab Powder: The Evaluation of Spray and Freeze-Drying for Production

**DOI:** 10.1371/journal.pone.0163109

**Published:** 2016-10-05

**Authors:** Gaurav Kanojia, Rimko ten Have, Arjen Bakker, Koen Wagner, Henderik W. Frijlink, Gideon F. A. Kersten, Jean-Pierre Amorij

**Affiliations:** 1 Intravacc (Institute for Translational Vaccinology), Bilthoven, The Netherlands; 2 University of Groningen, Department of Pharmaceutical Technology and Biopharmacy, Groningen, The Netherlands; 3 Leiden Academic Centre of Drug Research, Drug Delivery Technology, Leiden, The Netherlands; 4 AIMM Therapeutics, Amsterdam, The Netherlands; Kermanshah University of Medical Sciences, ISLAMIC REPUBLIC OF IRAN

## Abstract

In prospect of developing an oral dosage form of Infliximab, for treatment of Crohn’s disease and rheumatoid arthritis, freeze-drying (vial vs Lyoguard trays) and spray-drying were investigated as production method for stable powders. Dextran and inulin were used in combination with sucrose as stabilizing excipients. The drying processes did not affect Infliximab in these formulations, i.e. both the physical integrity and biological activity (TNF binding) were retained. Accelerated stability studies (1 month at 60°C) showed that the TNF binding ability of Infliximab was conserved in the freeze-dried formulations, whereas the liquid counterpart lost all TNF binding. After thermal treatment, the dried formulations showed some chemical modification of the IgG in the dextran-sucrose formulation, probably due to Maillard reaction products. This study indicates that, with the appropriate formulation, both spray-drying and freeze-drying may be useful for (bulk) powder production of Infliximab.

## Introduction

Therapeutic antibodies are among the most important biopharmaceuticals. Therapeutic proteins in liquid may require a cold chain during storage and transport as they may be prone to physical and chemical degradation. In order to improve their stability, therapeutic proteins are often dried by methods such as freeze-drying or spray-drying [1, 2]. By removal of water, the protein stability increases as degradation pathways and protein mobility are reduced in the dried state.

In the pharmaceutical industry, freeze-drying is the most commonly used method, to dry therapeutic antibodies [3]. Generally, freeze-drying is performed using vials. After freezing, ice is removed by sublimation (primary drying stage) and water is removed by desorption (secondary drying stage) [4–8]. Lyoguard trays have been developed for drying bulk volumes of liquid into bulk powder that can easily be collected and processed further by cryo-milling [9]. A maximum of 1800 ml of liquid can be dried in one Lyoguard tray. The tray top is made of semipermeable membrane that permits water vapor to pass through. The membrane is not permeable to microorganisms and therefore sterility of the product being dried is maintained. [4, 10]. Although extensive research has been carried out on lyophilization of monoclonal antibody (mAbs) in vials [2, 11–13], there is little published data on freeze-drying of pharmaceuticals in Lyoguard trays [14].

Spray-drying on the other hand starts with the nebulization of a liquid. The droplets are dried via evaporation in a continuous airflow. The evaporation cools the droplets, preventing high-temperature-exposure of the product [8, 15–17]. The shear stress generated during nebulization may affect the product quality but can be minimized by addition of excipients [18].

In order to successfully dry antibodies and especially to retain their biological activity during drying and subsequent storage, stabilizing excipients are required. Saccharides or polyols are the most commonly used stabilizers for drying of proteins [11, 19, 20]. A number of studies have reported that sugars are thought to provide the stabilization effect by forming a sugar glass matrix that reduces diffusion and molecular mobility of proteins thereby protecting the biological product [21, 22]. Moreover, during the drying process the hydrogen bonds between the water molecules and protein are replaced by bonds with the hydroxyl groups of the sugar, thereby maintaining the structural integrity of the protein [23, 24]. Non-reducing sugars, like sucrose and trehalose are widely used excipients. Moreover, polysaccharides like inulin and dextran have been described as excipients that can increase the temperature (glass transition temperature, T_g_) above which the glass to rubber transition of the dried powder may take place [25].

In this study, Infliximab is formulated using sucrose and the polysaccharides inulin or dextran in order to produce dry powder bulk material by spray-drying or freeze-drying. Infliximab is a mAb against TNF-α and used in the treatment of inflammatory bowel diseases (IBD). Infliximab neutralizes the effects of Tumor Necrosis Factor-α by binding to the soluble and transmembrane forms and inhibits binding to its receptors and thus suppressing the inflammatory cascade [26]. In the commercial Infliximab products (e.g. Remicade or Inflectra), the mAb is formulated using sucrose (50 mg/mL), phosphate buffer and Tween 80 (0.05 mg/mL) and freeze-dried [19].

The objective of this study was to compare two drying methods, freeze-drying (vial vs Lyoguard trays) and spray-drying to obtain stable dried Infliximab formulations. Freeze-drying in Lyoguard trays and spray drying are of interest because of the potential to scale up the drying process for bulk powder production. This would facilitate the development of an oral [27] or other dosage form as alternative for the current intravenous administration route [28]. Since Infliximab acts in the gut, systemic uptake is not necessary. Depending on the route of delivery, additional research would be required for secondary processing. This study focusses on excipients and excipient combinations for production of thermostable Infliximab powder formulations.

## Materials and Methods

### Excipients

The following materials were used: sucrose (Sigma-Aldrich), polysorbate 80 (Tween 80, Sigma-Aldrich), inulin (4kD; Sensus, Roosendaal), dextran (5kD; Pharmacosmos Denmark), sodium dihydrogen phosphate (Sigma-Aldrich), disodium hydrogen phosphate (Sigma-Aldrich) and 5 mg/mL of Infliximab antibody bulk (5 mg/ml in 5% sucrose and 0.005% (polysorbate 80, pH 7.2) (obtained from AIMM therapeutics).

### Infliximab production and purification

Recombinant infliximab was produced in CHO K1SV cells (Lonza). Infliximab was purified from CHO supernatants using Mab Sure-resin (GE Healthcare) and gel filtration (Superdex200, GE Healthcare). Purified antibodies were formulated in disodium hydrogen phosphate (1.2 mg/mL) adjusted to pH 7.2 with sodium dihydrogen phosphate (0.25 mg/mL), 5% sucrose and 0.005% polysorbate80.

### Infliximab formulations

The formulation buffer consists of disodium hydrogen phosphate (1.2 mg/mL) adjusted to pH 7.2 with sodium dihydrogen phosphate (0.25 mg/mL).

Three formulations were prepared for drying (Table 1).

1. **Sucrose based:** final concentration of 5% (50 mg/mL) sucrose, 0.005% Tween 80 (polysorbate 80) and 5 mg/mL of Infliximab antibody.
2. **Sucrose-Inulin based:** final concentration of 5% (50 mg/mL) sucrose, 6% (60 mg/mL) inulin, 0.005% Tween 80 (polysorbate 80) and 5 mg/mL of Infliximab antibody.
3. **Sucrose-Dextran based:** final concentration of 5% (50 mg/mL) sucrose, 6% (60 mg/mL) dextran, 0.005% Tween 80 (polysorbate 80) and 5 mg/mL of Infliximab antibody.

**Table 1:** Formulations classified based on excipients and drying methods.

Drying Method	Formulation type		
	sucrose	sucrose-inulin	sucrose-dextran
Freeze-dried			
-Vials	F1	F2	F3
-Lyoguard tray	L1	L2	L3
Spray-dried		S1	S2

These formulations were, lyophilized in glass vials (F), lyophilized in Lyoguard trays (L) or spray dried (S) resulting in dried formulations. The sucrose based formulation was not spray dried. The sucrose based formulation acts as a control for the licensed product. Furthermore, the study focused on sugar excipients (inulin and dextran) which have a high glass transition temperature and were anticipated to render a thermostable powder.

### Freeze-drying (FD)

#### Drying in glass vials

Vials filled with 1 mL (11 mL vial, Nuova Ompi, Italy) of the three formulations F1, F2 and F3 were loaded into a pilot freeze-dryer (Zirbus, The Netherlands) and subsequently frozen to −40°C by reducing the shelf temperature at a rate of 1°C/min. The vials were kept at a temperature of −40°C for 2 h. For the primary drying phase, the shelf temperature was increased at a rate of 0.2°C/h to −35°C (while decreasing the chamber pressure to 0.05 mbar) followed by drying for 96 h. The secondary drying phase was performed by further increasing the shelf temperature at a rate of 0.02°C/min to 25°C at a chamber pressure of 0.01 mbar, followed by 24 h drying at 25°C. At the end of the cycle, the vials were closed under vacuum, sealed with alu-caps and kept at 4°C until analysis.

#### Drying in Lyoguard trays

The Lyoguard trays (LGT2000, Gore & associates, Tilburg, The Netherlands) [4] were filled with 30 mL, 15 mL and 15 mL of the three formulations L1, L2 and L3. Filled Lyoguard trays were loaded into the freeze dryer and dried according to the same protocol as used for vials (as described above).

### Spray drying (SD)

A lab scale B-290 spray dryer was employed in conjunction with a high performance cyclone and a B-295 dehumidifier (Büchi Labortechnik AG, Flawil, Switzerland) using a closed loop configuration. Infliximab bulk powders were produced from 30 mL solutions of formulation S1 and S2 respectively. Production of the powders was performed using the following process settings: inlet temperature of 120°C, liquid feed flow rate of 1.0 mL/min, atomizing airflow of 667 L/h, and an aspirator flow rate was of 22 m^3^/hr (the maximum instrument capacity).

### Powder collection and further handling

In order to minimize exposure to environmental moisture all powder handling after drying was performed in a glove box (Terra Universals, series 100 plastic glove box, Fluerton CA, USA). The relative humidity (RH) was maintained below 3.0% with nitrogen purge in the environment, and was monitored using an RH sensor. Spray dried powders were filled in vials and sealed in glove box. The vials were stored at 2–8°C until further analysis. For accelerated stability, study samples were stored at 60°C for 1 month. The choice of this temperature was based on the melt curve determined by differential scanning fluorimetry for Infliximab liquid samples (data not shown). The beginning of the melt of–CH2 domain occurs around 60°C. The liquid infliximab stored at this temperature served as the negative control. Moreover, it is known that infliximab in dried product is not affected at lower temperatures of 40°C [27].

### Residual moisture content analysis (RMC)

The residual moisture content (RMC) was determined using a Water Detection System 400 (Sartorius, Göttingen, Germany). The principle of the analysis is based on as a combination of a thermal gradient and water detection by gas coulometry using a sensor described elsewhere [29]. The sample (approximately 20 mg) was introduced in small pans into the oven chamber. The temperature program included an initial rise of temperature to 100°C and maintained for 3 minutes. It was further raised to 130°C and maintained for 2 minutes. It was raised further to 180°C in 3 minutes. The software from the equipment calculated the final RMC values. All RMC analysis were performed in triplicate (1 vial for each test, 3 vials in total).

### Sample reconstitution and protein concentration determination

The dried Infliximab samples were reconstituted in ultrapure water by gentle swirling, followed by filtration with a 0.22 μm syringe filter (Whatman). Some sucrose-inulin formulated Infliximab samples showed some insoluble material after reconstitution, those samples were centrifuged (1 min, 16k rpm) and supernatant was used for analysis. The protein concentration in the reconstituted samples was measured spectrophotometrically, using a Nanodrop 1000 spectrophotometer (Thermo Scientific). The reconstituted samples were stored at 4°C.

### Size exclusion chromatography

Size exclusion chromatography (SEC) was performed to measure the percentage of soluble protein aggregates and degradation products. For non-stressed reconstituted Infliximab, 500 μL of solution containing 1.0 mg IgG was injected on a prep-grade HiPrep Superdex200 16/600 column (GE Healthcare), equilibrated with Infliximab formulation buffer. PBS was used as the mobile phase. For temperature stressed Infliximab, 10 μL of 1.0 mg/mL (diluted in PBS) antibody was injected on an analytical Sephax Zenix-C SEC300 gel filtration column (Sepax, Delaware, USA), equilibrated with 150 mM NaPO_4_, pH 7. The choice of second column was based on the rationale that it has a higher separation efficiency (5–1250 kDa) for separating aggregates compared to HiPrep Superdex (10–600 kDa). During elution, the absorbance was measured by a UV-detector set at a wavelength of 215 nm and 280 nm. The percentage of aggregation was calculated using UNICORN 5.0 software (GE Healthcare).

### Sodium dodecyl sulfate polyacrylamide gel electrophoresis (SDS-PAGE)

To determine possible antibody fragmentation, non-reducing and reducing SDS-PAGE was performed using a 4–20% mini Protean TGX gradient gel (BIORAD). Reconstituted Infliximab samples, containing 2.0 μg mAb, were diluted into 20 μL of XT SDS-PAGE sample buffer (Biorad, The Netherlands). When indicated samples were reduced, DTT (Biorad, The Netherlands) was added to a final concentration of 5 mM. Samples were incubated at 95°C for 5 minutes before loading on the gel. Dual color protein standard (Biorad, The Netherlands) was used as a molecular weight ladder. Protein bands were visualized by staining the gels with BioSafe Coomassie stain (Biorad, The Netherlands) and destained in ultrapure water. Stained gels were imaged on a GelDoc imager (Biorad, The Netherlands).

### IgG ELISA

The IgG content of the reconstituted samples was determined with an ELISA. The 96-well ELISA plate (Costar, #3590) was coated overnight at 4°C with polyclonal goat-anti-human-IgG-Fc (5 ug/mL in PBS; Jackson) 100 μL per well. The plate was washed three times with wash-buffer (PBS containing 0.05% Tween 80) followed by blocking for 2 hours with blocking buffer (1% m/v fish skin gelatin, Roche). The control and reconstituted Infliximab samples were filtered with a 0.22 μm syringe filter (Whatman) and a dilution series in blocking buffer with a starting concentration of 500 ng/mL, diluted to 1 ng/mL (according to Nanodrop measurement), was added to the plate. For stressed samples, a starting concentration of 1000 ng/mL was used. After 5x washing, 100 μL of detection antibody goat-anti-human-IgG HRP conjugate (Jackson) was added and incubated for 1 hour at room temperature. After 2 washing 5x, bound HRP-conjugate was detected with tetramethyl benzidine (TMB) as the peroxidase substrate. The enzymatic reaction was allowed to proceed at room temperature for 3 min and stopped by adding 50 μL of 1 M sulfuric acid. The absorbance was measured at a wavelength of 450 nm using an Envision plate reader (Perkin Elmer). Based on different dilutions, the data was calculated back to the theoretical concentration of 5 mg/mL.

### TNF-Capture ELISA

The TNF-binding capacity of Infliximab was quantified by a capture ELISA. The method in brief as follows: a 96-well ELISA plate was coated overnight at 4°C with mouse-anti-human TNFα capture antibody (1:400; Pelikan TNF kit, Abcam) 100 μL per well. The plate was washed three times with wash buffer (PBS containing 0.05% Tween80) followed by blocking for 2 hours with the blocking buffer (1% m/v fish skin gelatin, Roche). Later, 100 μL of solution containing 10 ng/mL in blocking buffer Human-rTNFα (Life Technologies) was added and incubated for 1h at room temperature. After washing, 100 μL of diluted Infliximab control in blocking buffer (500 ng/mL) and reconstituted samples were added to the corresponding wells. This was followed by addition of 100 μL of detection antibody–goat-anti-IgG HRP (Jackson) was added and incubated for 1h at room temperature. The bound antibody was detected with tetramethyl benzidine (TMB) as peroxidase substrate. The enzymatic reaction was allowed to proceed at room temperature for 3 min and stopped by adding 50 μL of 1 M sulfuric acid. The absorbance was measured at a wavelength of 450 nm using (an Envision plate reader (Perkin Elmer). The data is represented as actual concentration vs absorbance.

## Results and Discussion

In order to produce an Infliximab containing powder, different drying methods and excipient combinations were evaluated. In addition, an accelerated stability study was performed (at 60°C) to examine whether the thermal stability would be affected by the composition of the formulation.

### Antibody powder properties

#### After drying and storage at 2–8°C

Spray-drying (SD) and freeze-drying (FD) in Lyoguard trays were investigated as drying methods for production of dried Infliximab formulations. In order to compare with the standard drying method used for current marketed Infliximab products, each Infliximab formulation was also freeze-dried in vials. Independent of the drying method used, the RMC of all but one formulations were below 3% (Table 2), the recommended RMC limit for dried biologicals [30]. The exception was the RMC of freeze-dried sucrose formulation (F1). A lower residual moisture content is preferred since an increased water content in the dried formulation may affect the protein stability, for example through aggregate formation caused by the plasticizing effect of water (lower glass transition temperature and subsequent increase in molecular mobility). The selected SD and FD processes were suitable for producing dried Infliximab product within the prescribed RMC limits for the inulin and dextran containing formulations. The product obtained from FD may require a cryo-milling step [9] to obtain powder form. In addition, it was found that drying in Lyoguard trays resulted in a higher RMC than drying in vials, except for the sucrose-based formulation. The increase in RMC could probably be due to the reduced moisture removal caused by the membrane barrier.

**Table 2:** Residual moisture content of dried Infliximab formulations.

Drying Method	RMC of Infliximab Formulation (%)		
Freeze-dried	Sucrose	sucrose-inulin	sucrose-dextran
-Vials	3.9 ± 0.3	1.1 ± 0.1	1.2 ± 0.1
-Lyoguard tray	2.6 ± 0.2	2.4 ± 0.3	2.5 ± 0.3
Spray-dried	-	1.2 ± 0.1	1.5 ± 0.2

**Fig 1:**
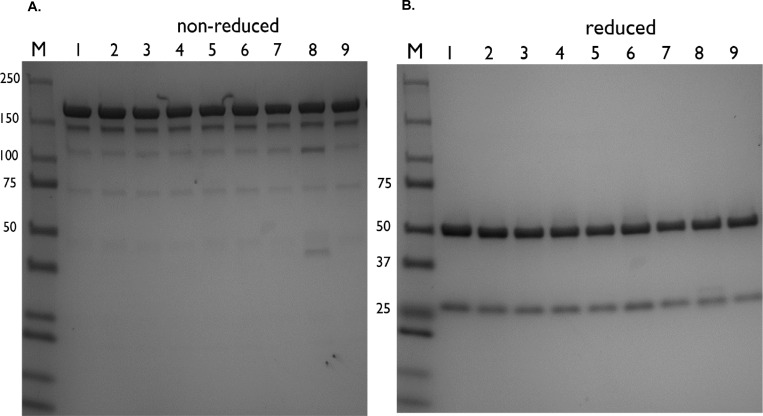
A. T = 0 Non-reducing SDS-PAGE analysis of reconstituted Infliximab samples. M: Dual color protein standard, 1: commercial Infliximab, 2: Infliximab (AIMM), Freeze-dried (vials) 3: sucrose-based (F1), 4: sucrose-inulin (F2), 5: sucrose-dextran (F3), Freeze-dried (Lyoguard trays) 6: sucrose-inulin (L2), 7: sucrose-dextran (L3), Spray–dried 8: sucrose-inulin (S1), 9: sucrose-dextran (S2). B.T = 0 Reducing SDS-PAGE of reconstituted Infliximab samples. M: Dual color protein standard, 1: commercial Infliximab, 2: Infliximab (AIMM), Freeze-dried (vials) 3: sucrose-based (F1), 4: sucrose-inulin (F2), 5: sucrose-dextran (F3), Freeze-dried (Lyoguard trays) 6: sucrose-inulin (L2), 7: sucrose-dextran (L3), Spray–dried 8: sucrose-inulin (S1), 9: sucrose-dextran (S2).

Analysis of non-dried Infliximab and reconstituted Infliximab by size exclusion chromatography (SEC) showed the presence of intact IgG without degradation products for all samples (data not shown). This interpretation was confirmed by SDS-PAGE analysis (see Fig 1A and 1B). Fig 1A shows the expected IgG band at around 150 kDa under non-reduced conditions and antibody fragments at 50 kDa (IgG1 heavy chain) and 25 kDa (kappa light chain) under reduced conditions (see Fig 1B, reduced samples). This demonstrates that the drying methods did not affect the Infliximab backbone. Thus, all samples consist of intact monomeric IgG, irrespective of the applied drying process or formulation. In addition, the IgG content of the reconstituted dried Infliximab formulations measured by the Nanodrop spectrophotometer was found to be in line with the theoretical concentration (data not shown). Apparently, the insoluble material observed in Inulin containing formulations did not contain any protein.

#### After accelerated stability storage—1 month at 60°C

All Infliximab samples were incubated at a temperature of 60°C for 1 month. At these extreme conditions, liquid Infliximab formulated with sucrose and Tween 80 readily aggregated and precipitated. Storage of liquid Infliximab at 60°C for 3 days results in visible aggregation. The filtrate was analyzed for IgG content and less than 1% was recovered.

**Fig 2:**
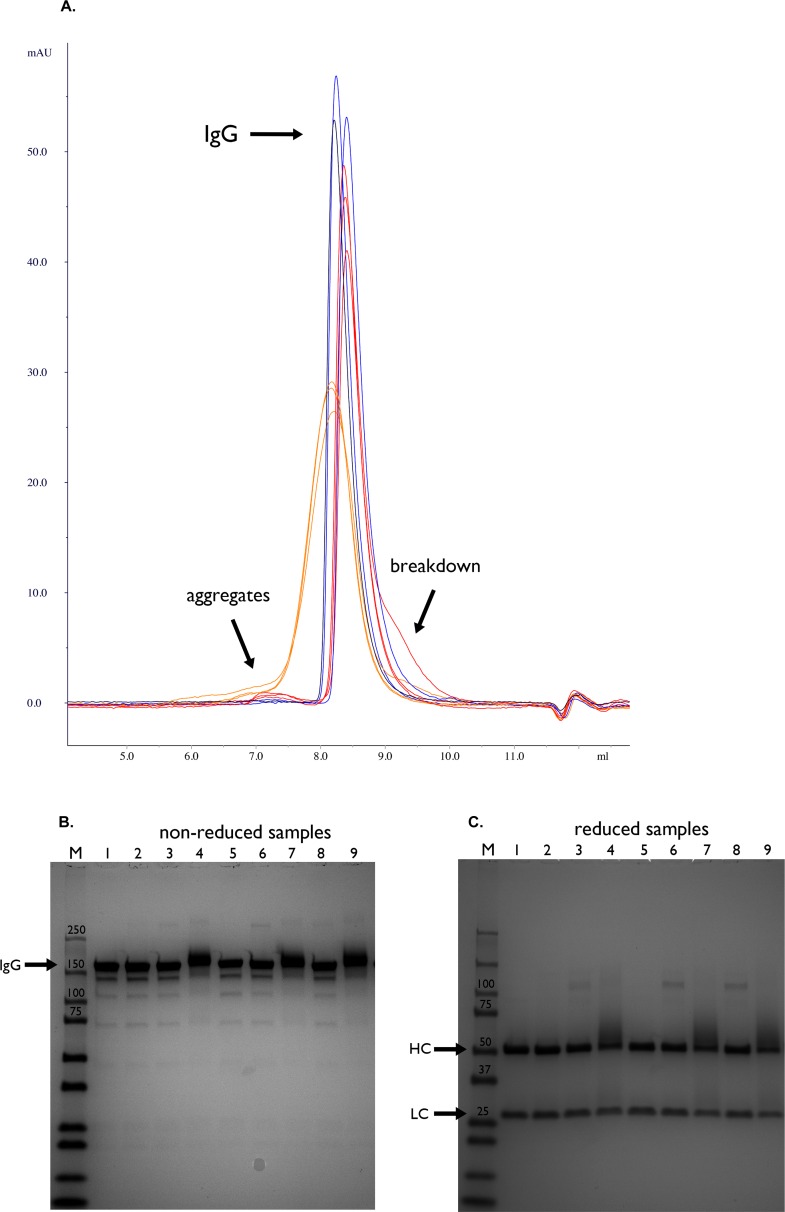
A. T = 1 month (60°C) gel filtration runs of reconstituted Infliximab samples. Commercial Infliximab (Remicade) stored at 4°C shown in black. Samples dried in sucrose based buffer shown in blue. Samples dried with sucrose-inulin excipients shown in red. Samples dried with sucrose-dextran based excipient shown in orange. B. T = 1 month (60°C) Non-reducing SDS-PAGE analysis of reconstituted Infliximab samples stored at 60°C. M: Dual color protein standard, 1: commercial Infliximab (stored at 4°C), Freeze-dried (vials) 2: sucrose-based (F1), 3: sucrose-inulin (F2), 4: sucrose-dextran (F3), Freeze-dried (Lyoguard trays) 5: sucrose-based (L1), 6: sucrose-inulin (L2), 7: sucrose-dextran (L3), Spray–dried 8: sucrose-inulin (S1), 9: sucrose-dextran (S2). C. T = 1 month (60°C) Reducing SDS-PAGE analysis of reconstituted Infliximab samples stored at 60°C. M: Dual color protein standard, 1: commercial Infliximab (stored at 4°C, Freeze-dried (vials) 2: sucrose-based (F1), 3: sucrose-inulin (F2), 4: sucrose-dextran (F3), Freeze-dried (Lyoguard trays) 5: sucrose-based (L1), 6: sucrose-inulin (L2), 7: sucrose-dextran (L3), Spray–dried 8: sucrose-inulin (S1), 9: sucrose-dextran (S2).

The SEC chromatograms showed the presence of small amounts of IgG degradation/ reaction products in all powders (Fig 2A). The sucrose formulated Infliximab formulations showed least aggregation (see Table 3). In spray-dried sucrose-dextran powder, aggregation was most evident.

**Table 3:** The percentage of antibody aggregates determined by SEC present in samples after storage at 60°C for 1 month. In case of non-stressed sample, material was stored at 2–8°C.

Formulation Type	Method	Aggregation (% of total)
Liquid	(non-stressed)	0.0
Sucrose	F1	0.2
	L1	0.2
Sucrose-inulin	F2	0.7
	L2	1.2
	S1	1.1
Sucrose-dextran	F3	0.6
	L3	1.1
	S2	2.6

There was also evidence for adduct formation as concluded from the broadened IgG peak from SEC analysis in the formulation with dextran. The dextran-sucrose formulations showed an increase in molecular weight of the IgG as well as smearing of the antibody band, indicating heterogeneous chemical addition (Fig 2B, lanes 4, 7, 9). The reducing SDS-PAGE analysis indicated modifications to the heavy chain in the dextran-sucrose based formulations, as evidenced by smearing of the protein band at 50 kDa (Fig 2C, lane 4, 7 and 9). These findings may be explained by the occurrence of IgG-dextran adducts formation by the well-known Maillard reaction [31]. Inulin-sucrose based formulations show extra bands at around ±100 kDa (Fig 2C, lane 3, 6 and 8) suggesting a possible dimerization via two heavy chain crosslinks. Inulin contains reducing groups [32] and can react with amino groups of proteins resulting in possible crosslinking. This observation was limited to storage at elevated temperatures. One would not suspect such extent of modifications during storage at lower temperatures (<25°C).

### Preservation of Biological activity

#### After drying and storage at 2–8°C

**Fig 3:**
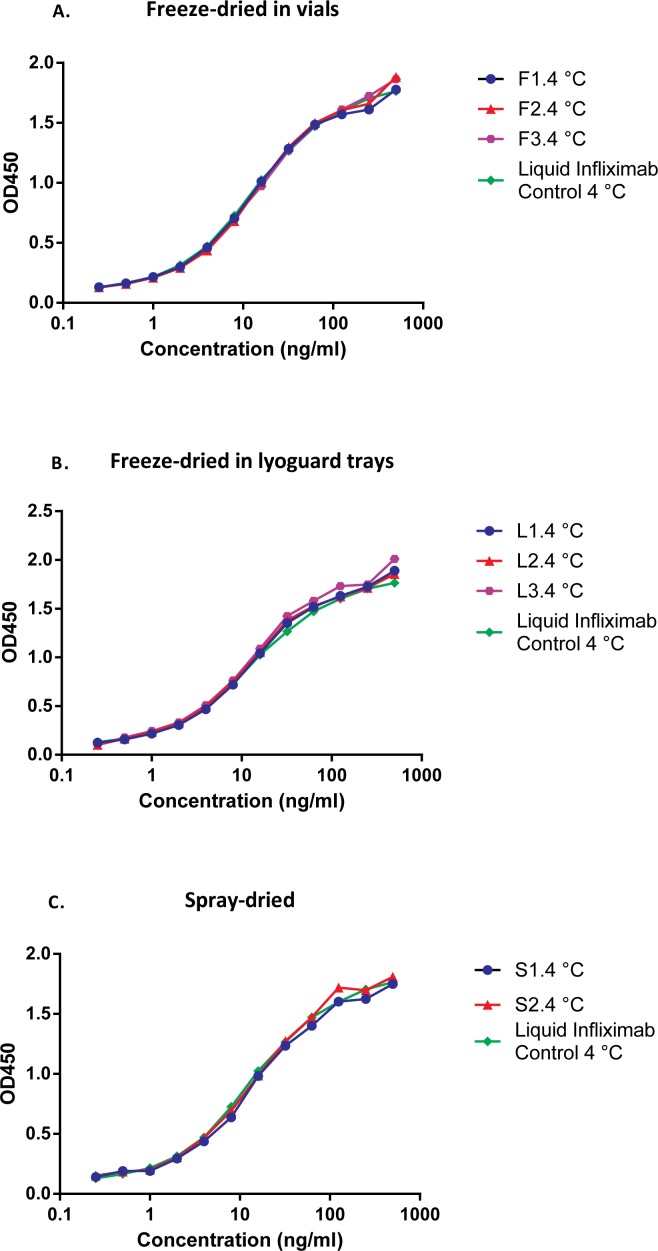
A. T = 0 TNF binding assay of reconstituted freeze-dried in vials Infliximab formulations. Control: Infliximab (AIMM, stored at 4°C), Freeze-dried (vials): sucrose-based (F1), sucrose-inulin (F2) and sucrose-dextran (F3). B. T = 0 TNF binding assay of reconstituted freeze-dried in Lyoguard trays Infliximab formulations. Control: Infliximab (AIMM, stored at 4°C), Freeze-dried (Lyoguard trays): sucrose-inulin (L2) and sucrose-dextran (L3). C.T = 0 TNF binding assay of reconstituted spray-dried Infliximab formulations. Control: Infliximab (AIMM, stored at 4°C), Spray-dried: sucrose-inulin (S1) and sucrose-dextran (S2).

The biological activity of Infliximab was assessed by determining its TNF binding ability. There was a minimal difference between the untreated control and Infliximab powders immediately after drying (Fig 3). This is in accordance with SEC and SDS-page analysis. Obviously, the drying method and formulation composition hardly affected the antibody’s ability to bind to TNFα. This was also in line with the IgG recovery of freshly dried samples when compared to the theoretical value (Table 4).

**Table 4:** The IgG concentrations observed by IgG ELISA for Infliximab samples.

Formulation Type	Method	Theoretical IgG concentration (mg/mL)	IgG concentration(mg/mL)Day 0	IgG concentration (mg/mL)Day 30 at60°C
Sucrose	F1	5.0	4.3 ± 0.04	4.3 ± 0.07
	L1	5.0	5.1 ± 0.05	4.6 ± 0.10
Sucrose-Inulin	F2	5.0	4.5 ± 0.06	4.2 ± 0.06
	L2	5.0	5.6 ± 0.02	4.0 ± 0.25
	S1	5.0	4.6 ± 0.04	2.4 ± 0.27
Sucrose-Dextran	F3	5.0	4.4 ± 0.05	3.6 ± 0.09
	L3	5.0	5.1 ± 0.05	4.8 ± 0.04
	S2	5.0	4.6 ± 0.06	2.3±0.05
Infliximab (AIMM)	Liquid control	10.0	9.17 ± 0.03	0.06 ± 0.007 (day 7)

#### After accelerated stability storage—1 month at 60°C

**Fig 4:**
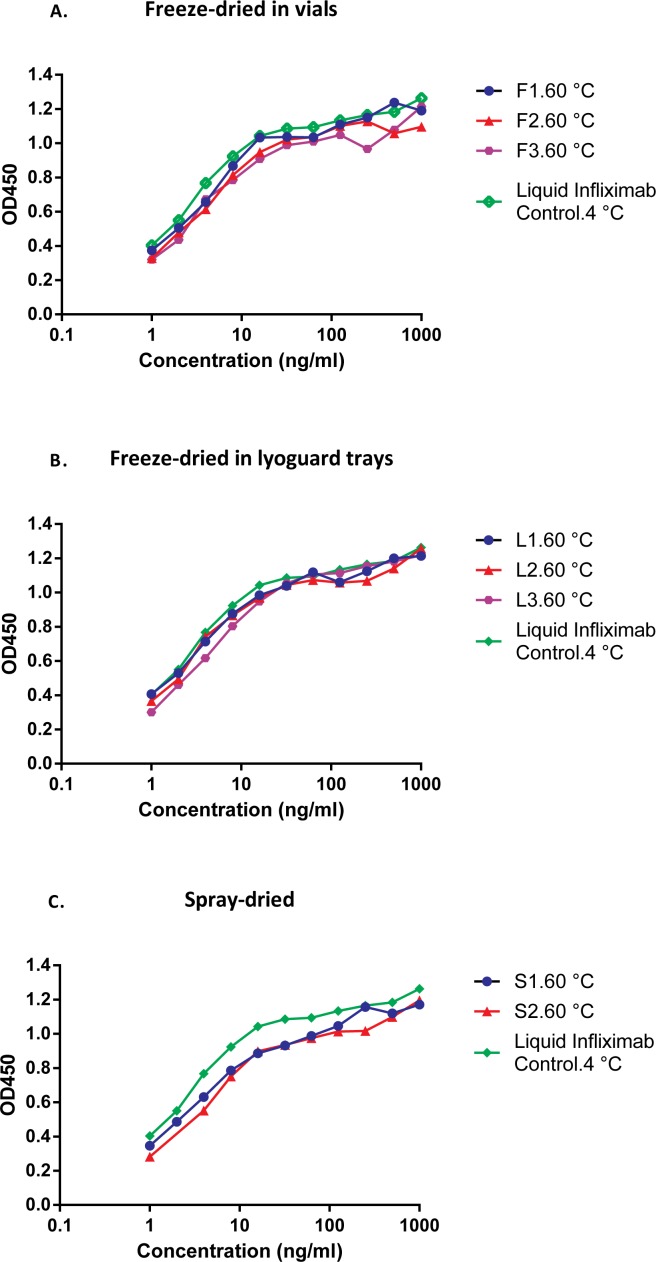
A.T = 1 month (60°C) TNF binding assay of reconstituted freeze-dried in vials Infliximab formulations after storage for 1 month at 60°C. Control: Infliximab (AIMM, stored at 4°C), Freeze-dried (vials): sucrose-based (F1), sucrose-inulin (F2) and sucrose-dextran (F3). B.T = 1 month (60°C) TNF binding assay of reconstituted freeze-dried Lyoguard trays Infliximab formulations after storage for1 month at 60°C. Control: Infliximab (AIMM, stored at 4°C), Freeze-dried (Lyoguard trays): sucrose-inulin (L2) and sucrose-dextran (L3). C. T = 1 month (60°C) TNF binding assay of reconstituted spray-dried Infliximab formulations after storage for1 month at 60°C. Control: Infliximab (AIMM, stored at 4°C), Spray–dried: sucrose-inulin (S1) and sucrose-dextran (S2).

Spray-dried Infliximab formulations showed a decreased TNF binding ability after storage at elevated temperatures (Fig 4C). This observation was in line with the reduced IgG recovery of both spray-dried Infliximab samples after storage at 60°C (see Table 4). Surprisingly, no such reduction in IgG recovery or TNF binding ability (Fig 4A and 4B) was noted in the freeze-dried formulations stored at 60°C, which is remarkable since both Lyoguard samples had a higher RMC than the spray-dried samples.

## Concluding Remarks

Freeze-drying and spray-drying were shown to be successful methods for producing Infliximab powders, using three different sugar combinations for drying. The monoclonal antibody retained its physical stability and TNF binding ability during the drying process. Compared to the liquid formulation, drying clearly improved the stability of the Infliximab. Spray-dried formulations showed a reduced thermostability (at 60°C) compared to the freeze-dried products as concluded from aggregate formation. Both aggregates and Maillard reaction products are unwanted because these may increase the potential risk of clinical immunogenicity [33, 34]. A real time stability study might be useful to examine whether formation of these reaction products is also relevant at temperatures below 25°C.
